# Epidemiology of anti-tuberculosis drug resistance in a chinese population: current situation and challenges ahead

**DOI:** 10.1186/1471-2458-11-110

**Published:** 2011-02-17

**Authors:** Yan Shao, Dandan Yang, Weiguo Xu, Wei Lu, Honghuan Song, Yaoyao Dai, Hongbing Shen, Jianming Wang

**Affiliations:** 1 Department of Chronic Infectious Diseases, Jiangsu Provincial Center for Disease Prevention and Control, Nanjing, PR China; 2 Department of Epidemiology and Biostatistics, School of Public Health, Nanjing Medical University, Nanjing, PR China

## Abstract

**Background:**

Drug resistance has been a cause of concern for tuberculosis (TB) control in both developed and developing countries. Careful monitoring of the patterns and trends of drug resistance should remain a priority.

**Methods:**

Strains were collected from 1824 diagnosed sputum smear positive pulmonary TB patients in Jiangsu province of China and then tested for drug susceptibility against rifampicin, isoniazid, ethambutol and streptomycin. The prevalence and patterns of drug resistance in mycobacterium tuberculosis (MTB) isolates were investigated. Multiple logistic regression analysis was performed to identify the risk factors for multidrug resistant (MDR) bacterial infection. The strength of association was estimated by odds ratio (OR) and 95% confidence interval (95% CI).

**Results:**

The drug susceptibility tests showed that 1077(59.05%) MTB strains were sensitive to all the four antibiotics and the other 747(40.95%) strains were resistant to at least one drug. The proportions of mono-drug resistance were 28.73% for isoniazid, 19.41% for rifampicin, 29.33% for streptomycin, and 13.98% for ethambutol, respectively. The prevalence of MDR-TB was 16.61%, which was significantly different between new cases (7.63%) and those with previous treatment history (33.07%). Geographical variation of drug resistance was observed, where the proportion of MDR-TB among new cases was higher in the central (9.50%) or north part (9.57%) than that in the south area (4.91%) of Jiangsu province. The age of patients was significantly associated with the risk of drug resistance (P < 0.001) and the adjusted OR (95% CI) was 1.88(1.26-2.81) for patients aged 35-44 years when compared with those 65 years or older. Patients with previous treatment history had a more than 5-fold increased risk of MDR-TB (adjusted OR: 6.14, 95% CI: 4.61-8.17), compared with those previously not having been treated.

**Conclusions:**

The high prevalence of drug resistance has been a major challenge for TB control. Prevention and control of drug-resistant TB should be emphasized by the revised DOTS (direct observed therapy, short course) program through prompt case detection, routine and quality-assured drug susceptibility test for patients at high risk of resistance, programmatic treatment with both first and second-line medicines, and systematic treatment observation, with priority for high MDR-TB settings.

## Background

Tuberculosis (TB) is a leading cause of death in humans due to an infectious agent (mycobacterium tuberculosis, MTB) and it remains a major public health burden in developing countries [1]. Globally, there were an estimated 8.9-9.9 million incident cases of TB in 2008, most of which occurred in Asia and Africa, with the first two countries being India and China [2]. The current anti-tuberculosis therapies are fraught with problems, predominantly because of the long-term treatment and the increasing occurrence of drug resistance [3]. Drug resistance can be simply defined as the capacity of organisms and their progeny to remain viable or to multiply in the presence of the concentration of the drug that would normally destroy or inhibit cell growth [4]. Drug resistance of MTB was reported soon after the introduction of anti-tuberculosis drugs in the last century and now has been a critical threat to global TB control [5]. One of the particularly dangerous form is multidrug-resistant (MDR) TB, which is defined as resistance to at least isoniazid and rifampicin, the two most powerful anti-tuberculosis drugs, because the treatment of such cases is more complex, lengthy, expensive, frequently less successful and usually produce more severe adverse reactions [6]. In 2008, an estimated 390,000-510,000 cases of MDR-TB have emerged globally [7]. China reported a high proportion of drug resistance with the highest burden of cases in the world, just following the countries of the former Soviet Union [8]. It was estimated that the prevalence of MDR-TB was 5.7% among new cases and 25.6% among those previously treated in China, which confirmed previous estimates that about 100,000 MDR-TB cases emerged annually [7].

China has reached the global targets for case detection and treatment success now, but careful monitoring of the patterns and trends of drug resistance should remain a priority. In order to obtain insights into the prevalence and distribution of anti-tuberculosis drug resistance, China joined the global surveillance project organized by WHO/IUATLD (World Health Organization/International Union against Tuberculosis and Lung Diseases). By 2007, 13 out of 31 provinces (without Jiangsu) in China have been involved in this surveillance system [9]. As a province located along the eastern coast of China, Jiangsu covers an area of 102.6 thousand square kilometres and contains 13 municipalities and 106 counties (districts), with a total population of 77 million in 2009. DOTS (direct observed therapy, short course) strategy for TB has been introduced in Jiangsu in the 1990s and been 100% available at the county level by now [10]. However, there are still great challenges facing TB control, particularly for the early detection and effective treatment. Unfortunately, until now, no study has been conducted to investigate the current situation and patterns of drug resistance among TB patients at the provincial level in Jiangsu.

Therefore, the aim of the present study was to assess the drug susceptibility patterns of MTB in Jiangsu and explore potential risk factors for MDR-TB in order to provide policy-makers with recommendations for better-organized TB control programs.

## Methods

### Study population

The sampling method referred to the "Guidelines for surveillance of drug resistance in tuberculosis" developed by WHO/IUATLD [11]. Thirty counties (districts) from Jiangsu province were selected systematically and the sample size was estimated based on the following parameters: (1) The annual reported new sputum smear positive (SS+) cases were 23603 and previously treated SS+ cases were 5524 (based on the surveillance data in Jiangsu province); (2) The precision was set at 2% for new cases and 4% for previously treated cases; (3) The initial mono-drug resistance rate was set at 6% among new cases and 16% among previously treated cases based on the proportion of rifampicin resistant isolates from a pilot study with small sample size (unpublished); Additionally, the sample size was amplified by taking into account the design effect of the cluster sampling method and potential no-response of study subjects. Finally, the estimated sample size was 2035, which included 1247 new cases and 788 previously treated cases. Sixty-seven SS+ patients from each site were recruited continuously as expected, including 41 new patients and 26 previously treated patients. The period of case recruitment was set between May 1, 2008 and December 31, 2008. All newly registered pulmonary TB patients with sputum smear positive tests in selected study sites were eligible for inclusion. Eligible patients should be continuously recruited since May 1, 2008, until the sampling site reaches a minimum number of cases.

### Data collection

After obtaining informed consent, a standard questionnaire was completed for each recruited patient to collect demographic data and the history of treatment. Cases were classified into new and previously treated ones based on patient's self-reports or available medical documents. The definitions of new/previously treated cases referred to the WHO guidelines [7,11]. A 'new case' was defined as a newly registered episode of TB in a patient who, in response to direct questioning, denied having had any prior anti-tuberculosis treatment (for up to one month), and in study sites where adequate documentation was available, for whom there was no evidence of such history. A 'previously treated case' was defined as a newly registered episode of TB in a patient who, in response to direct questioning admitted having been treated for TB for one month or more, or, in study sites where adequate documentation was available, there was evidence of such history.

### Laboratory test

Three sputum smear samples were collected from each subject with labelled plastic bottles. The Ziehl-Neelsen hot staining method was used for sputum smear microscopy test. The two sputum samples with the highest bacterial counts were cultured, and one culture was submitted for drug susceptibility test (DST). Sputum smear microscopy and culture were performed at the level of county (district) laboratory while DST was done at the provincial reference laboratory. The sputum samples were decontaminated with 4% sodium hydroxide (NaOH), centrifuged, and then cultured on Lowenstein-Jensen (LJ) culture media. The LJ culture media were incubated at 37°C and observed on days 3 and 7 to detect contaminations and/or fast growth of atypical mycobacteria and subsequently every week to note the growth and the morphology of the colonies. Identification of MTB was done using the p-nitrobenzoic acid (PNB) and thiophene carboxylic acid hydrazine (TCH) resistance test. Growth in LJ medium containing PNB indicates that the bacilli do not belong to the MTB complex. Species other than MTB were excluded from the current analysis. DST utilizes the same type of LJ medium and inoculation methods as culture techniques. LJ medium was impregnated with isoniazid (INH), rifampicin (RIF), streptomycin (SM), and ethambutol (EMB) according to the proportion method as recommended by WHO/IUATLD [8,11]. The concentrations of anti-tuberculosis drugs were 0.2 μg/ml for INH, 40 μg/ml for RIF, 4 μg/ml for SM, and 2 μg/ml for EMB. The growth of colonies in the drug-containing plate was compared to the control plate as a proportion. If the bacterial growth on the medium with the specific drug was ≥1% compared to the control, the strain was declared resistant to the specific drug; or it was defined as sensitive when the growth rate was < 1% compared to the control. MDR-TB was defined as isolates being resistant to at least RIF and INH. Internal and external quality controls were conducted through the whole study period. For internal quality assurance on DST, a standard H37Rv laboratory strain was included for each batch of culture. External quality control for sputum smear microscopy and culture was conducted by the provincial TB reference laboratory in Jiangsu and further undertaken by the supranational reference laboratory of Hong Kong.

### Data analysis

Data were double entered with EpiData 3.1 (Denmark) and discrepancies were checked against the raw data. The prevalence of mono- and multi- drug resistance among new and previously treated TB cases was calculated. Multiple logistic regression analysis was performed to identify the risk factors for MDR bacterial infection. Variables included in the final model were chosen based on the biological plausibility as well as statistical and epidemiologic criteria. The strength of association was estimated by odds ratio (OR) and 95% confidence interval (95% CI). All tests of significance were two sided and a significant threshold was set at 0.05. All analyses were performed using the STATA statistical software (version 10.0; StataCorp, College Station, TX).

### Ethical consideration

This project has been approved by Institutional Review Board of Nanjing Medical University. Written informed consent was obtained from all participants. Ethics has been respected throughout the whole study period.

## Results

**Figure 1:**
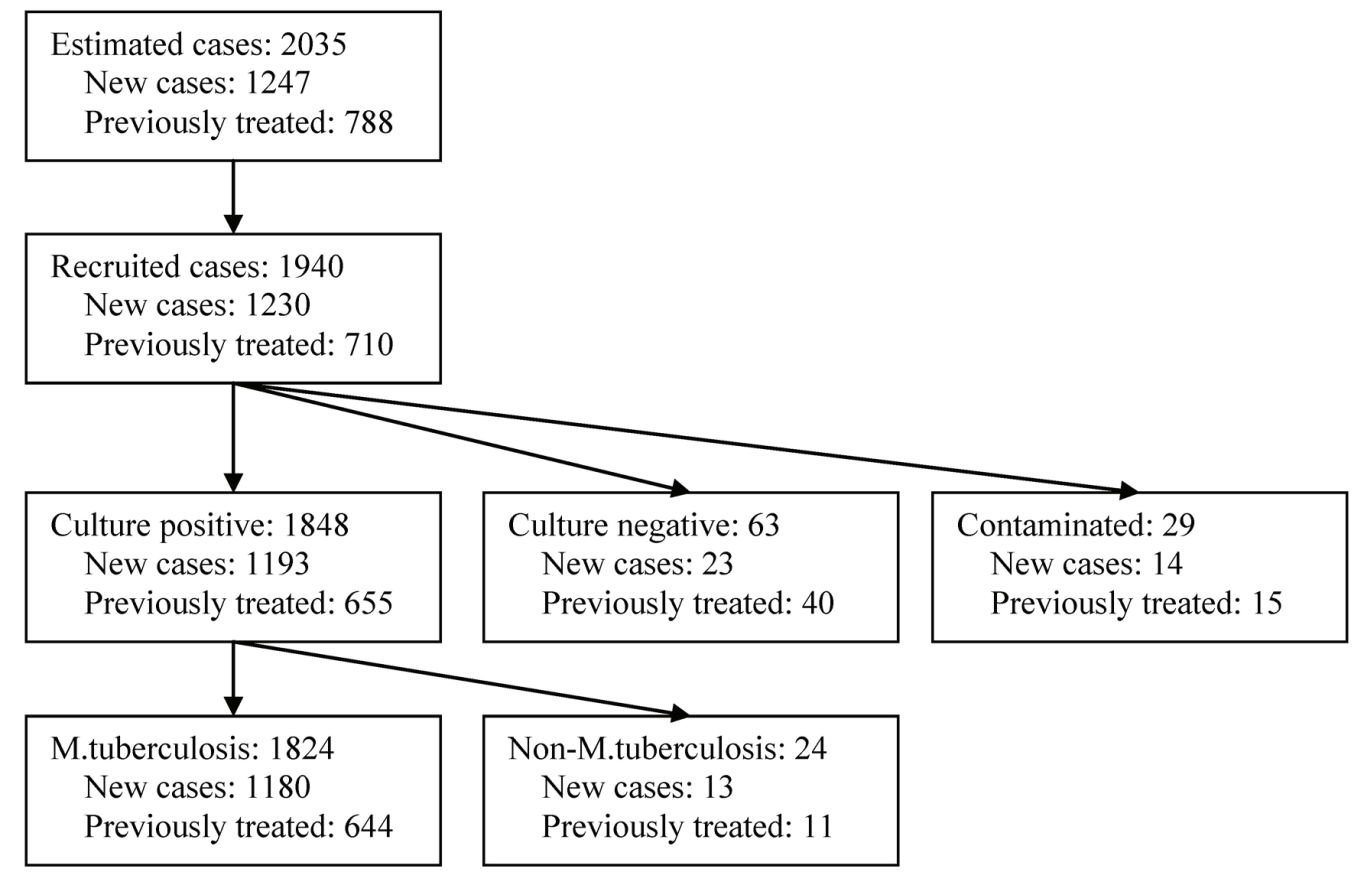
**Flow chart of the study design**.

**Figure 2:**
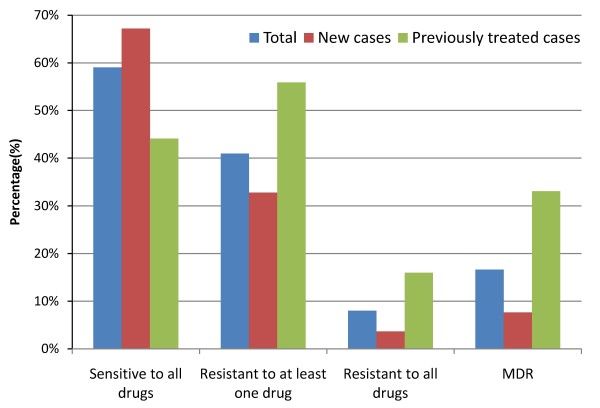
**Percentage of drug resistance by treatment history**. Drug susceptibility was tested against rifampicin, isoniazid, ethambutol and streptomycin. MDR: resistant to at least isoniazid and rifampicin.

**Figure 3:**
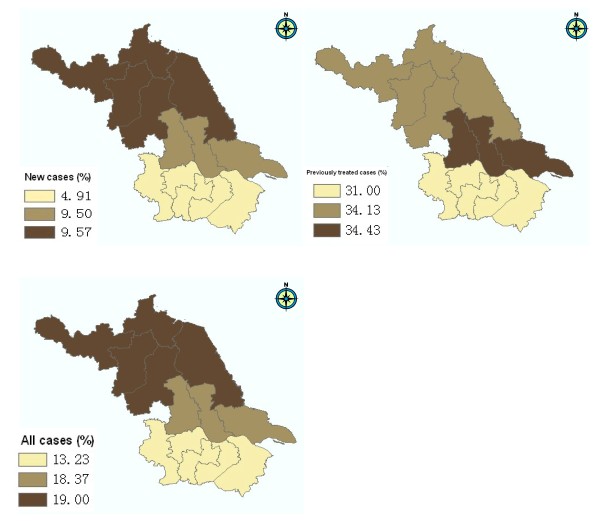
**Geographical variation of MDR-TB in Jiangsu province**. The study sites were categorized as three groups (north, central and south) based on their locations. MDR: resistant to at least isoniazid and rifampicin.

**Figure 4:**
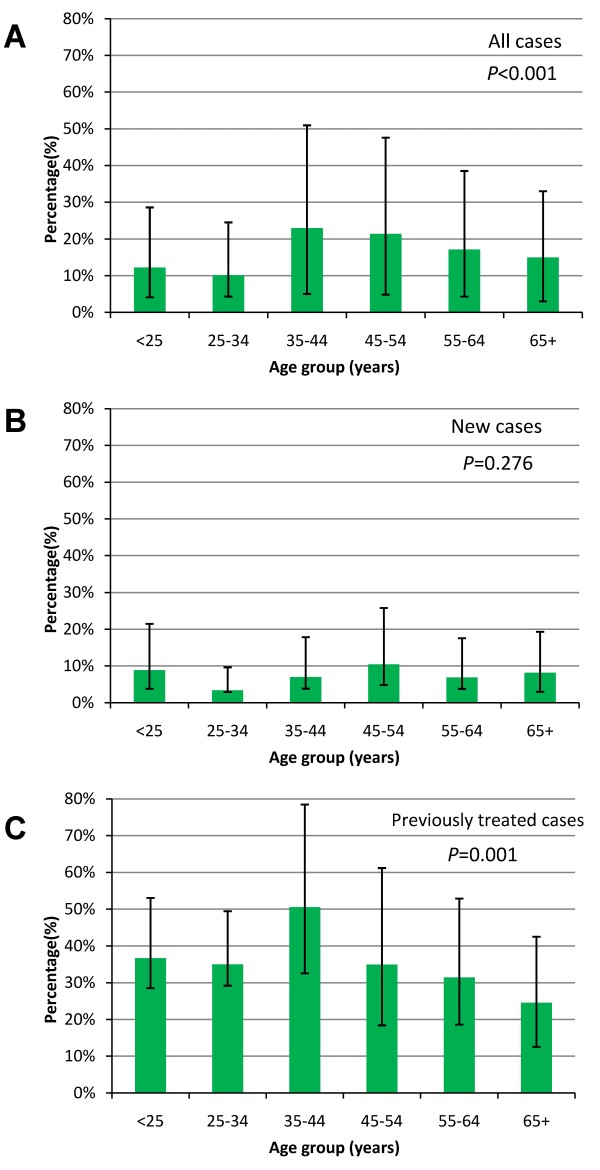
**Percentage of MDR-TB by age group**. A. Percentage of MDR-TB by age group among all cases. B. Percentage of MDR-TB by age group among new cases. C. Percentage of MDR-TB by age group among previously treated cases. MDR: resistant to at least isoniazid and rifampicin. *P *values were estimated based on the Chi-square test.

Among 1940 sputum smear positive TB patients recruited in the study, 1848 (95.26%) cases were positively cultured, 63 (3.25%) cases were negatively cultured, and 29 (1.49%) cases were contaminated. After excluding 24 cases infected with non-tuberculosis mycobacterium, 1824 cases determined as MTB were involved in the final analysis (Figure 1). Most of them (97.81%) were Han Chinese. The nationalities of remaining patients included Hui, Mongolian, Miao, Yi, Zhuang, Tu, Buyi, Bai, and Hani etc. The average age was 50.8 ± 19.3 years and the sex ratio was 2.7 with 1340 (73.46%) men and 484 (26.54%) women. Among them, 1180 (64.69%) were new cases and 644 (35.31%) were those previously treated. The DST results showed that 1077 (59.05%) strains were sensitive to all first-line antibiotics tested in our study and 747 (40.95%) were resistant to at least one drug. The proportions of mono-drug resistance were 28.73% for INH, 19.41% for RIF, 29.33% for SM, and 13.98% for EMB, respectively (Table 1). Individual or combined drug resistance to INH, RIF, EMB and SM was higher in previously treated cases than that in new cases. The details of multi-drug resistance are presented in Table 2. Among all isolates, 303(16.61%) strains were resistant to both INH and RIF (MDR-TB), and 146(8.00%) strains were resistant to all four first-line anti-tuberculosis drugs (Table 2, Figure 2). The proportion of MDR-TB was 33.07% in previously treated cases, which was significantly higher than that (7.63%) in new cases (χ^2 ^= 194.76, *P *< 0.001) (Table 2, Figure 2). We further divided the study sites into three groups (south, central and north) based on their locations in Jiangsu and found the geographical variation of MDR-TB risk estimates. The proportions of MDR were 13.23% in south, 18.37% in central and 19.00% in north area of Jiangsu, respectively (Table 3). The geographical difference was statistically significant among new cases (Figure 3). As shown in table 3, a higher proportion of MDR-TB in new cases was observed in the central or north part but not in the south area. The percentage of MDR-TB grouped by age was illustrated in Figure 4. The frequency of MDR-TB was much higher in young adults and peaked at 35-44 years old. Compared with patients aged 65 years or older, the adjusted OR (95% CI) was 1.88(1.26-2.81) for those aged 35-44 years.

**Table 1:** Observed mono-drug resistance among tuberculosis cases in Jiangsu Province, China

Category	Cases	Sensitive	Drug resistant^†^				
			
			At least one drug	INH	RIF	EMB	SM
		n(%)	n(%)	n(%)	n(%)	n(%)	n(%)
Total	1824	1077(59.05)	747(40.95)	524(28.73)	354(19.41)	255(13.98)	535(29.33)
New cases	1180	793(67.20)	387(32.80)	228(19.32)	112(9.49)	97(8.22)	287(24.32)
Previously treated cases	644	284(44.10)	360(55.90)	296(45.96)	242(37.58)	158(24.53)	248(38.51)

†: isoniazid (INH); rifampicin (RIF); streptomycin (SM); ethambutol (EMB)

**Table 2:** Observed multi-drug resistance among tuberculosis cases in Jiangsu Province, China

Category	Cases	Drug resistant^†^				MDR^‡^
			
		HR	HRE	HRS	HRSE	
		n(%)	n(%)	n(%)	n(%)	n(%)
Total	1824	37(2.03)	26(1.43)	94(5.15)	146(8.00)	303(16.61)
New cases	1180	9(0.76)	5(0.42)	33(2.80)	43(3.64)	90(7.63)
Previously treated cases	644	28(4.35)	21(3.26)	61(9.47)	103(15.99)	213(33.07)
*χ^2^*		26.95	23.87	37.98	86.29	194.76
*P*		**< 0.001**	**< 0.001**	**< 0.001**	**< 0.001**	**< 0.001**

†: HR: isoniazid+rifampicin; HRE: isoniazid+rifampicin+ethambutol;

HRS: isoniazid+rifampicin+streptomycin;

HRSE: isoniazid+rifampicin+streptomycin+ethambutol

‡: MDR, resistant to at least isoniazid and rifampicin

**Table 3:** Observed drug resistance in different areas of Jiangsu Province, China

Area of residence	New cases			Previously treated cases			Total		
			
	Cases	Resistant^†^	MDR^‡^	Cases	Resistant^†^	MDR^‡^	Cases	Resistant^†^	MDR^‡^
		n(%)	n(%)		n(%)	n(%)		n(%)	n(%)
South	489	132(26.99)	24(4.91)	229	125(54.59)	71(31.00)	718	257(35.79)	95(13.23)
Central	221	82(37.10)	21(9.50)	122	72(59.02)	42(34.43)	343	154(44.90)	63(18.37)
North	470	173(36.81)	45(9.57)	293	163(55.63)	100(34.13)	763	336(44.04)	145(19.00)
*χ^2^*		12.76	8.76		0.65	0.69		13.11	9.84
*P*		**0.002**	**0.012**		0.723	0.708		**0.001**	**0.007**

†: Resistant to at least one drug; ‡: MDR, resistant to at least isoniazid and rifampicin.

Table 4 showed the number of patients with MDR-TB and the ORs of potential risk factors. Patients with previous treatment history had a more than 5-fold increased risk of MDR-TB (adjusted OR: 6.14, 95% CI: 4.61-8.17), compared with those previously not having been treated. The risk of MDR-TB for patients living in the central or north part is more than 1.4-times of that for those living in the south part, with the ORs of 1.47 (95% CI: 1.01-2.13) and 1.42 (95% CI: 1.05-1.92), respectively (Table 4). No significant association was found between cigarette smoking and the risk of MDR-TB (OR: 1.01, 95% CI: 0.72-1.42).

**Table 4:** Factors associated with multi-drug resistant tuberculosis

Factors	Cases	No-MDR^†^	MDR^†^	cOR(95% CI) ^‡^	*P*	aOR(95% CI) *	*P*
	n = 1824	n = 1521, n(%)	n = 303, n(%)				
**Sex**							
Men	1340	1129(84.25)	211(15.75)	1		1	
Women	484	392(80.99)	92(19.01)	1.26(0.96-1.65)	0.099	1.43(0.99-2.06)	0.058
**Age (years)**							
65+	547	465(85.01)	82(14.99)	1		1	
55-64	298	247(82.89)	51(17.11)	1.17(0.80-1.72)	0.418	1.11(0.74-1.67)	0.602
45-54	276	217(78.62)	59(21.38)	1.54(1.06-2.24)	**0.022**	1.53(1.03-2.28)	**0.035**
35-44	270	208(77.04)	62(22.96)	1.69(1.17-2.44)	**0.005**	1.88(1.26-2.81)	**0.002**
25-34	188	169(89.89)	19(10.11)	0.64(0.38-1.08)	0.095	0.87(0.49-1.53)	0.626
< 25	245	215(87.76)	30(12.24)	0.79(0.51-1.24)	0.306	1.38(0.84-2.29)	0.206
**Treatment history^#^**							
No	1180	1090(92.37)	90(7.63)	1		1	
Yes	644	431(66.93)	213(33.07)	5.99(4.57-7.84)	**< 0.001**	6.14(4.61-8.17)	**< 0.001**
**Area of residence**							
South	718	623(86.77)	95(13.23)	1		1	
Central	343	280(81.63)	63(18.37)	1.48(1.04-2.09)	**0.029**	1.47(1.01-2.13)	**0.044**
North	763	618(81.00)	145(19.00)	1.54(1.16-2.04)	**0.003**	1.42(1.05-1.92)	**0.023**
**Cigarette smoking^&^**							
Never	862	706(81.90)	156(18.10)	1		1	
Ever	954	809(84.80)	145(15.20)	0.81(0.63-1.04)	0.098	1.01(0.72-1.42)	0.942

†:MDR, multidrug-resistant, resistance to at least isoniazid and rifampicin; ‡: cOR, crude odds ratio; *aOR, adjusted odds ratio, adjusted for sex, age, treatment history, area and cigarette smoking; #:Those having previously received anti-tuberculosis treatment for more than one month was defined as previously treated cases; &: 8 cases with missing value.

## Discussion

As a public health dilemma, drug resistance has been an obstacle to achieve the goal of effective global TB control. Recently, the emergence of MDR and XDR (extensively drug-resistant) TB has incurred a very alarming challenge to global health [5,12]. Appreciating the importance of documenting the drug susceptibility of MTB, WHO has implemented a worldwide surveillance programme while findings showed regional and national variations in the magnitude and trends of drug-resistance [13]. The high prevalence of MDR recently reported from the expanding number of provinces surveyed in China and Russia is indicative of a larger epidemic than previously suspected [6]. In recent years, the substantial structural transformation of China's economy has contributed to the world's largest ever-peacetime flow of migration. Mobility of population has been proved to strengthen the transmission dynamics of TB as well as antimicrobial drug-resistant organisms [14,15]. As a more developed eastern province, Jiangsu absorbed a large proportion of migrations within China. Now it has been an area with the highest population density (753 per square kilometre) among all provinces of China. Exploring the prevalence and pattern of drug resistance could substantially facilitate the local and countrywide TB control.

### Role of age and sex

In most countries, the majority of TB patients are male [16]. However, male or female TB patients could have different levels of risks for drug resistance due to differences in access to health-care services or exposure to other risk factors [7]. In the present study, the OR of harboring MDR-TB strains for female was 1.43 (95% CI: 0.99-2.06) compared with male, showing a borderline association between MDR-TB and the sex of patients. Discovering gender disparities associated with the risks of MDR-TB could provide insight into the development of targeted measures and improve access to health care and reduce the risk of acquiring drug-resistance. The association between age and the risk of MDR-TB is not well established in the literatures as different studies use different cut-off points for age groups. MDR-TB patients were more likely to be younger than 65 years [17,18]. A systematic review analysis in Europe revealed the pooled risk of MDR-TB for people younger than 45 was higher than that among older patients (OR: 1.52, 95% CI: 1.13-2.03) [17]. In this study, we found that the frequency of MDR-TB was much higher in young adulthoods and peaked at 35-44 years old, which was similar to the findings from MDR-TB surveillance data in 13 countries of Central and Eastern Europe (CEEUR) [7]. This pattern suggests that MDR-TB epidemic in Jiangsu is a relatively recent phenomenon and bears the highest toll on young adults. We assumed that age-related difference in treatment adherence might be a possible explanation, as patients at 35-44 years old were often occupied by study, work or other activities on a daily basis, in contrast with the more sedentary lifestyle of old patients [18].

### Effect of previous anti-tuberculosis treatment

Drug resistance in MTB isolates arises from spontaneous genetic mutations and can be amplified through selection pressure and further exaggerated by poor adherence of patients [5]. Drug-resistant TB is either acquired due to poor management of treatment or transmission from infectious drug-resistant TB patients [19]. As found in many other studies, the history of anti-tuberculosis treatment has been consistently associated with the risk of MDR-TB [20]. A systematic review on 29 studies in Europe reported the pooled risk of MDR was up to 10 times higher in previously treated cases than in never treated ones [17]. Our study is consistent to the former reports that revealed the importance of previous treatment history in the risk of drug resistance. These previously treated patients often constitute a very heterogeneous group including those who experience relapse after receiving successful treatment, those who return after default, and those who start receiving a re-treatment regimen after having experienced previous treatment failure [21].

### Geographical variation

Geographical variation of drug resistance was observed in the present study, where a higher proportion of MDR-TB among new cases was found in the central or north part as compared with that in the south area. Understanding the patterns of transmission is important in TB control because acquired resistance and primary resistance require different control strategies [22]. To prevent primary drug resistance, new technologies for early distinguishing drug resistance and adopting effective measures to block the transmission are important. To reduce acquired drug resistance, development of high-quality drugs and strength of patient management are needed [22]. Lower proportion of primary MDR in the south part indicates that TB control in this region is rather successful and lacks substantial transmission of MDR strains as compared with the central and north part of Jiangsu. However, similar higher proportions of acquired drug resistance among TB patients in these three regions also cause concerns for strengthening drug development and patient management. Another interesting topic in the transmission of MDR-TB is the role of migration, as Jiangsu is a province absorbing a large number of non-permanent residents. In this study, we did not specifically design questions to distinguish the moving population from the native population. However, one question might be associated with this topic: "How long have you been living here"? We analyzed this variable, but found no significant association. One possible explanation might be a large part of moving population preferring to return to their home when they were suspected as TB. A revised epidemiological design is needed to further analyze the role of migration on MDR.

### Limitation of this study

Some sources of bias could exist in this study. First, there is a potential bias in estimation of drug resistance in previously treated cases. Although we interviewed the patients, reviewed the medical records and screened the case notification system to check previous episodes of TB, we could not exclude the possibility of misclassification. Second, we did not perform DST for second-line anti-tuberculosis drugs in this study. Thus, the prevalence of XDR is unclear. Third, there are two most useful sampling strategies for the surveillance of drug resistance: 100% sampling of diagnostic centres and cluster sampling [11]. Cluster sampling methods were adopted reasonably in the situations in which it was logistically difficult to cover the entire area and where the number of TB diagnostic centres is high. Cluster sampling is commonly used, rather than simple random sampling, mainly as a means of saving money when, for example, the population is spread out, and the researcher cannot sample from everywhere. The loss of effectiveness by the use of cluster sampling, instead of simple random sampling, should not be neglected. Though in this study, the sample size was multiplied by the design effect, the higher sampling error caused by the cluster sampling method should be taken into account.

### Impact

The importance of knowing the susceptibility to MTB has become more significant because of the increasing resistance rates, high travel activity of hosts and the limited available anti-tuberculosis agents. Surveillance is a crucial intermediate step to allocate resources and design treatment regimens. However, such population-based surveys are temporary measures until we know the drug-susceptibility profile of each patient, which can be used for prescription of individualized therapy. DST needs to be scaled up so that patients infected with susceptible MTB strains can get DOTS, those with mono-drug resistance can get intensive DOTS to prevent multidrug resistance, and those with MDR can get the life-saving therapy they need [23]. Unfortunately, routine DST of MTB is not available at the country level of China while the laboratories responsible for diagnosing cases have not been strengthened to meet these requirements, despite the drug resistance has captivated the attentions in the past decade. Even in some health facilities with necessary equipments, the results of susceptibility test still take more than several weeks, when the patients with MDR-TB have already been treated with INH and RIF for quite a long time. Therefore, new strategies, rapid diagnostic tools [24,25] (such as molecular line-probe assay), new anti-tuberculosis therapies, and effective vaccines are urgently needed [26]. A better understanding of the risk factors would help to determine groups of patients who would be more likely to have MDR-TB and thus prioritize the use of rapid diagnostic methods for the identification and determination of drug resistance.

## Conclusions

In conclusion, the high prevalence of drug resistance in Jiangsu province has been a major challenge for TB control. Patients with previous treatment history are at higher risk for MDR-TB. Monitoring of drug-resistance should be enhanced by periodic surveys to assess trends and take correct actions when necessary. Prevention and control of drug-resistant TB should be emphasized by the revised DOTS program through prompt case detection, routine and quality-assured DST for those patients at high risk of resistance, programmatic treatment with both first and second-line medicines, and systematic treatment observation, with priority for high MDR-TB settings.

## Competing interests

The authors declare that they have no competing interests.

## Authors' contributions

YS, DY, WX, WL, and HS conceived the idea and implemented the field study and laboratory tests. DY, YD, HS and JW participated in the statistical analysis and drafted the manuscript. All authors read and approved the final manuscript.

## Pre-publication history

The pre-publication history for this paper can be accessed here:

http://www.biomedcentral.com/1471-2458/11/110/prepub
